# The Functional Response of a Generalist Predator

**DOI:** 10.1371/journal.pone.0010761

**Published:** 2010-05-27

**Authors:** Sophie Smout, Christian Asseburg, Jason Matthiopoulos, Carmen Fernández, Stephen Redpath, Simon Thirgood, John Harwood

**Affiliations:** 1 Scottish Oceans Institute & Centre for Research into Environmental and Ecological Modelling, University of St Andrews, St Andrews, United Kingdom; 2 Centre for Pharmaceutical Policy and Economics (CEPPE), University of Eastern Finland, Kuopio, Finland; 3 Instituto Español de Oceanografía, Vigo, Spain; 4 Aberdeen Centre for Environmental Sustainability, School of Biological Sciences, University of Aberdeen and Macaulay Institute, Aberdeen, United Kingdom; 5 The Macaulay Institute, Aberdeen, United Kingdom; University of Utah, United States of America

## Abstract

**Background:**

Predators can have profound impacts on the dynamics of their prey that depend on how predator consumption is affected by prey density (the predator's functional response). Consumption by a generalist predator is expected to depend on the densities of all its major prey species (its multispecies functional response, or MSFR), but most studies of generalists have focussed on their functional response to only one prey species.

**Methodology and principal findings:**

Using Bayesian methods, we fit an MSFR to field data from an avian predator (the hen harrier *Circus cyaneus*) feeding on three different prey species. We use a simple graphical approach to show that ignoring the effects of alternative prey can give a misleading impression of the predator's effect on the prey of interest. For example, in our system, a “predator pit” for one prey species only occurs when the availability of other prey species is low.

**Conclusions and significance:**

The Bayesian approach is effective in fitting the MSFR model to field data. It allows flexibility in modelling over-dispersion, incorporates additional biological information into the parameter priors, and generates estimates of uncertainty in the model's predictions. These features of robustness and data efficiency make our approach ideal for the study of long-lived predators, for which data may be sparse and management/conservation priorities pressing.

## Introduction

There is a growing realisation that management which considers only the dynamics of individual species is inadequate for conserving biodiversity [1],[2]. This has led to calls for an ecosystem-based approach to management [3] particularly in the fields of fisheries [4], forestry [5] and wildlife management. Attempts to develop suitable ecosystem models have struggled with technical problems and lack of data [6], [7]. As a result, focus has shifted towards the identification of relatively easily measured metrics that reflect the overall status of ecosystems [8]. These metrics are useful for monitoring the impacts of management but provide little biological insight. Dynamic ecosystem models are therefore still necessary if the potential effects of different management options are to be evaluated. Quantitative descriptions of the interactions between generalist predators and their prey are a key component of these models [9].

Such quantitative descriptions of the trophic links in biological communities are formulated mathematically using functional response models. For example, Holling [10] proposed the following equation for a system in which a predator preys on one type of prey, whose abundance can be given in terms of biomass or numbers and is denoted by ***q***. The consumption rate (***F***) by a single predator (biomass or items consumed per unit time) is
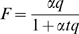
(1)Here ***t*** is the time taken by the predator to handle one item of prey. The parameter ***α*** represents the encounter rate between predators and prey that might be observed at very low prey density (i.e. the probability of a foraging predator encountering a prey item in one unit of time, given that the predator is searching throughout that time). Note that at these low prey densities, the predator does not spend any significant time handling prey items. At high prey density, the time spent by the predator in handling or consuming prey limits its consumption rate to ***1/t***.

Note that if prey abundance is known only by index ***q***, where ***q*** is proportional to the ‘true’ abundance of prey then we can write 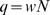, then
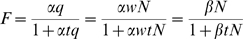
(2)Here, the parameter ***β*** incorporates a proportionality constant and can no longer be interpreted simply as an encounter rate.

This model can be further modified to allow for the possibility that encounter rates may change with prey abundance e.g. if predators are more likely to actively search for prey when that prey is abundant, or if prey have a refuge [11]. In one model this relationship is expressed as 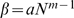 [12] [13] and we can then write
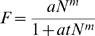
(3)Note here that when ***m = 1***, encounter rate is independent of prey density, 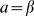, and equation (3) is equivalent to equation (2). When ***m>>>>1***, then encounter rate varies with prey density and ***a*** is a parameter that relates encounter rate to ***N^m−1^***. We refer to this as ‘the attack rate coefficient’, and this is distinct from the encounter rate 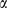 used in equation (1).

Eq. (3) is a versatile expression which can be simplified to correspond with several commonly-used FR equations [14]. If *m* = 1 and *t* = 0, we obtain a linear (Type 1) FR ; if *m* = 1 and t>0, we obtain a hyperbolic (Type 2) FR; and if *m*>1, the FR is sigmoidal (Type 3). Therefore, this simple formulation can potentially take into account biological complexity such as the use of refuge areas by prey, which can reduce encounter rates when prey density is low [15].

The form of the predator's functional response (the value of ***m***) may have important implications for prey populations. When ***m = 1*** (i.e. for a classic Holling type 2 functional response model) then at low densities of prey, the consumption rate ***F*** is proportional to prey abundance ***N*** (with ***a*** being the constant of proportionality). The per capita mortality rate for prey (or the probability that one prey item is taken in unit time) decreases as the prey become abundant because predators are fully ‘occupied’ in handling prey and cannot increase their consumption rate beyond the asymptotic limit ***1/t***. Therefore if prey that is initially abundant becomes scarce, while the population of predators remains constant, then the predation pressure on the remaining prey intensifies.

Where ***m>>>>1***, the predator-induced mortality has the potential to create a ‘predator pit’ for prey populations. It is still true that mortality of prey is reduced at high prey densities due to asymptotic predator consumption rates, and also true that predation pressure intensifies if abundant prey become scarce. However, at low prey densities, attack rates also decrease. It is then possible that prey mortality may be reduced at low prey density. As a result, it may be possible for prey to exist at low density in a ‘predator pit’. If the population increases about this level, predation pressure intensifies.

For a good explanation of these effects, including graphical explanation, see [16].

These arguments are developed under the assumption that the number of these specialist predators remains constant i.e. changes in prey mortality result only from the effects of the predator's functional response. However, if predator numbers change with prey density (i.e. if the predator shows a numerical response) then mortality rates will be affected and the consequences of predation for the prey population may be more complex [17].

Generalists consume more than one type of prey. We expect that consumption of any given prey (say, prey type 1) will depend on the availability of this and other prey in the system i.e. the mortality rate for prey type 1 will be reduced if an alternative prey type is present. Under the classic Holling functional response model, this effect is considered to be an outcome of the time spent by the predator in handling items of alternative prey which reduces the available time for encounters with prey 1. In the standard fomulations used in fishery models [12] the parameter ***a*** is often interpreted in terms of ‘suitability’ or ‘preference’. If we allow ***m>>>>1*** then preferences change with relative prey abundance [18].
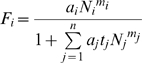
(4)Here, *n* is the number of prey species and all notation is now prey-specific [18], [19].

Because a generalist predator is able to adapt its diet and exploit different prey types, the dynamics of generalist predators are not necessarily tightly coupled to those of any one of their prey [20] and it is likely that the numerical response of predator populations will, like the functional response, be driven by the availability of more than one type of prey. Generalists may have dramatic effects on prey populations: they may dampen or eliminate cyclical interactions between specialist predators and their prey [21], hold prey populations at low density in predator pits [22] [23], drive rare species to extinction [24], [16], and help to prevent ecological meltdown [25]. The occurrence of these effects depends on the form of the predator's functional and numerical responses [26].

To model the role of generalist predators in community dynamics we need to describe their response to the abundance of **all** their prey species. Consumption rates are modelled using MSFRs [27]. However, there have been few attempts to fit MSFRs to field data because of the technical difficulties involved and because there is a widespread belief that no suitable data sets are available [28]. Here, we show how one type of MSFR can be fitted to field data on the consumption by a generalist predator (the hen or northern harrier *Circus cyaneus*) of its three most important prey species (the red grouse *Lagopus lagopus scoticus*, the meadow pipit *Anthus pratensis*, and the field vole *Microtus agrestis*) at 11 different combinations of prey density [29]. The red grouse is an economically important quarry in the UK, where hen harriers are classified as endangered species but are illegally killed [30] because of their perceived impact on grouse abundance [31].

## Methods

The methods of data collection for our study are fully explained in [32]. We summarise this information here to set the context for our statistical analysis, and then go on to present the statistical approach used to estimate the functional response parameters.

### The Data

We consider populations of hen harriers that breed on UK grouse moors. UK moorland is upland habitat, generally characterised by wet acidic soils and heather (*Calluna vulgaris*). Female hen harriers hunt over these areas throughout the year, although males often winter elsewhere. Harriers return to their breeding sites in spring and generally make their nests in tall heather [33]. Adult birds can consume a wide variety of prey but during the breeding season prey items delivered to nest for hen-harrier chicks tend to be dominated by meadow pipits, voles and red grouse chicks (adult grouse are rarely consumed during summer).

The data for this study were collected between 1992 to 1996 on 6 study moors in Scotland, and with additional data from a subset of sites in 1988. In total, 11 separate estimates of predation rates at different combinations of prey density were obtained. The methods used to obtain estimates of both predation rates and prey density are described briefly below. Details on the data collection are given in [34], [35].

Harrier diet was recorded by observers watching from hides set close to hen harrier nests over thousands of hours. The number and type of prey brought to the nest by parent birds was noted. Nests were observed during weeks 1 to 4 of the breeding season during which time it is estimated that 89% of prey items could be correctly identified [32]. Unidentified prey tended to be small and quickly consumed.

Prey density for the three major prey species of the hen harriers was estimated in each year. Red grouse chick density was estimated by transect sampling using pointing dogs with brood size and nest density estimated based on counts in June and July. Meadow pipits were counted by visual observation using line transect surveys. An index of field vole abundance was obtained from the numbers of voles caught per 100 snap-trap nights.

### Analysis

To provide a baseline for evaluating the implications of an MSFR, we first fitted a generalised single species functional response [13] to our data (equation (3)). Consumption of grouse by harriers is treated as a function of grouse density alone, and the presence of other prey in the system is ignored. *F* is the number of grouse chicks brought to a harrier nest per hour by individual parents, *N* is the density of grouse chicks in the area based on grouse nest counts and brood sizes.

Eq. (3) was fitted to the data using computer-intensive Bayesian methods (Monte Carlo Markov Chain –MCMC). We adopted this approach for three reasons. First, it allowed us to use a plausible sampling distribution for our response data, avoiding the need to assume normality or use transformations. Because the data were counts of predation events over fixed units of time and space, consumption was initially modelled by a Poisson sampling distribution around the fitted function. However, as is often the case with data of this kind [14], the residuals of the Poisson model were overdispersed. We therefore used a negative binomial sampling distribution, which includes an additional parameter for the degree of over-dispersion in the data [36]. Second, the Bayesian approach enabled us to incorporate independent information about the values for *t*, *a* and *m* in the form of prior distributions (more details of these distributions are given below). Third, the joint posterior distribution of the parameters could be directly approximated from the MCMC draws. This flexible approach to uncertainty has several advantages [37]. For example, we avoided the need for the kind of 2-stage fitting process that has previously been used to decide whether a functional response should be considered sigmoidal [38], because we represent uncertainty about the form of the functional response explicitly by the posterior distribution of the parameter *m*.

We then fitted a multispecies extension of eq. (4) in which consumption of any one prey type is a function of the availability of all types of prey. As in the single-species case, we assumed no observation error in the *N_j_* and individual negative binomial sampling distributions for the consumption rates *F_j_*. The correlation structure of the *F_j_* follows from eq. (2).

The MSFR is a non-linear function that employs as many response variables (the consumption of each prey species) as explanatory variables (the availability of each prey species). Not only does this impose apparently severe demands for data on prey availability and consumption, but there are few standard statistical techniques for fitting this kind of relationship. Those that are available cannot satisfactorily account for parameter and model uncertainty. However, Bayesian, computer-intensive methods place few restrictions on model structure, allowing us to apply a negative binomial sampling distribution to estimate the over-dispersion in the data and thus quantify parameter uncertainty rigorously and comprehensively for the multi-species case.

We used additional data and biological first principles to provide prior distributions for model parameters in both the single- and multi-species FR. For the *t_i_* we used a gamma prior with prey-specific mean and variance derived from published data. In our formulation of the FR, the attack rate on prey species *i* is given by 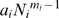. We used observational data on the attack rate for grouse [29] to derive a joint prior for *a_grouse_* and *m_grouse_*. Negative values of *m* are meaningless, and values of *m* between 0 and 1 imply that at unchanged abundance of other prey species, attack rate on one prey species can decrease with increasing density of this prey, which is implausible. We therefore chose a shifted gamma prior for *m* with minimum 1. The prior mean and variance of all *m_i_* were set to 2 and 0.9 respectively, giving a 95th percentile of 3.9. No prior knowledge was available for *a_pipit_* and *a_vole_*, so various relatively uninformative priors were used to check for robustness in the choice of prior. Results are shown for a gamma prior with mean 1 and variance 0.99.

MCMC was implemented with a Random Walk Metropolis-Hastings algorithm [39]. Variances of the proposal distributions were adjusted to achieve acceptance rates between 15–30% for each parameter. Plots of cumulative parameter means indicated that 4,000,000 iterations were satisfactory for convergence. We preceded these with a burn-in phase of 10,000 draws that did not contribute to the posterior. The validity of assuming a negative binomial sampling distribution for the consumption data was checked by comparing cumulative left probabilities for each datum, computed from its corresponding predictive distribution, with the negative binomial distribution (QQ plot).

## Results

Posterior point and interval parameter estimates for both the single- and multi-species FRs are shown in Table 1. For the single species FR (harriers preying on grouse only) the estimate of *m* was 1.09, implying a weakly sigmoidal FR. This is in contrast to the results of earlier work [32] which used non-linear least squares method and found support for a strongly sigmoidal FR. The difference is mainly due to our use of a less restrictive sampling distribution for the consumption data [40]. Also, the broad marginal posterior distribution of the parameter *t_grouse_* implies that the data do not support a well-defined asymptotic consumption rate for the single species FR. The mean estimate of *t_grouse_* implies an asymptotic consumption rate of approximately 3 items per hour for grouse chicks. However, it should be noted that this consumption rate is not predicted within the range of our observed prey densities: it is, in effect, an extrapolation and would only occur at levels of grouse density that are unrealistically high.

**Table 1:** Parameter values.

	Single-species functional response	Multi-species functional response
	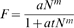	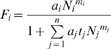
***a_grouse_***	0.00164 (0.000614–0.00243)	0.000673 (0.000484, 0.00119)
***a_vole_***	-	3.78 (2.20, 5.45)
***a_pipit_***	-	1.904 (0.941, 3.16)
***t_grouse_***	0.325 (0.0275–0.919)	2.74 (2.04, 3.46)
***t_vole_***	-	2.32 (0.960, 3.40)
***t_pipit_***	-	1.676 (1.39, 2.09)
***m_grouse_***	1.09 (1.00–1.31)	2.51 (2.33, 2.69)
***m_vole_***	-	1.14 (1.00, 1.44)
***m_pipit_***	-	1.18 (1.02, 1.41)

Mean parameter estimates for the single species functional response (middle column of table) and multi-species functional response (RHS column) fitted to the hen-harrier data set. The parameters are ***a***, the encounter parameter which relates prey density to attack rate, ***t***, the handling time (where ***1/t*** gives the maximum consumption rate), and ***m***, the shape parameter (values of ***m>>>1*** indicate that switching occurs). 95% Bayesian credible intervals are shown in brackets. Subscripts indicate the prey species for which each parameter was estimated.

There was no overlap between the posterior credible intervals for *t_grouse_* and *m_grouse_* obtained from the single-species FR and the MSFR (see Table 1). The mean estimates of *t_grouse_*, *t_pipit_ and t_vole_* respectively imply maximum consumption rates of 0.365, 0.597 and 0.431. Note that the handling time estimated for grouse under this model is much higher than that estimated under the single-species model, and consequently, the predicted maximum consumption rate for harriers feeding solely on grouse is lower (and probably more realistic). We also note that the handling times for the three prey species under the multi-species model are fairly similar to one another. This seems reasonable, given that any prey item must be carried back to the nest by the parent birds to be fed to the chicks no matter what its size.

**Figure 1:**
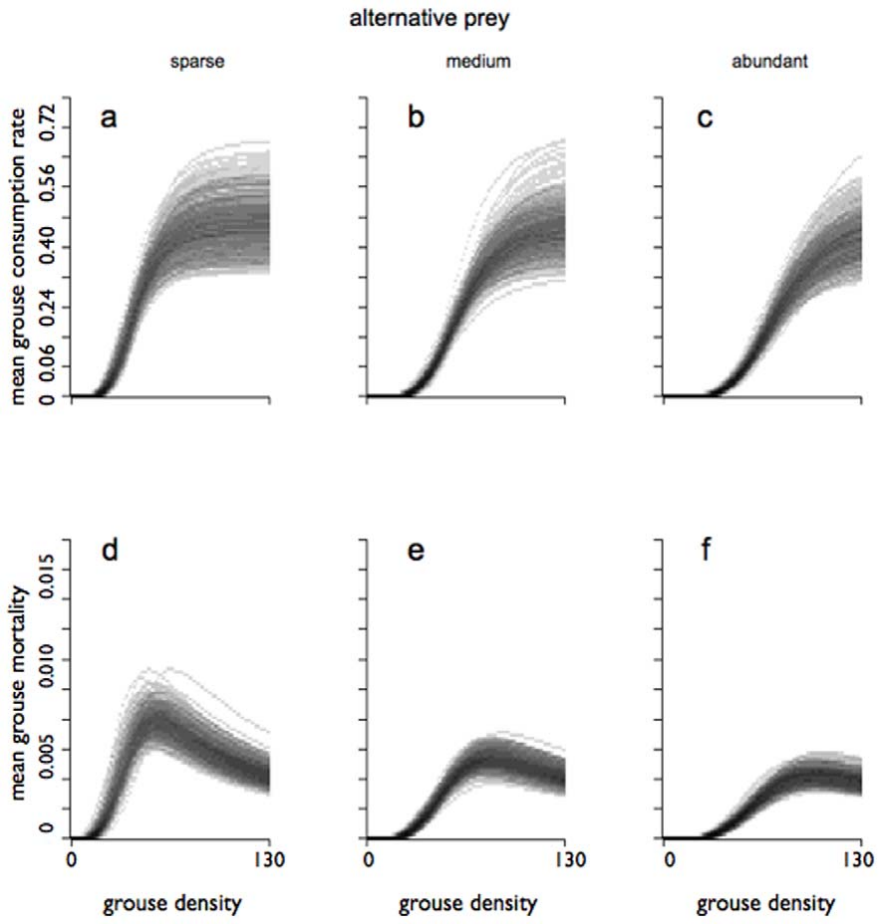
Estimated consumption rate and mortality rate for grouse chicks, as a function of grouse density. Estimated mean consumption is shown in the top row (a,b,c) and per capita mortality is shown in the bottom row (d,e,f), at various densities of alternative prey. Per capita grouse chick mortality was calculated as (hourly consumption rate)/(grouse chick density). The grey shades represent the posterior probability density at each point. In the left-hand column (a,d), both meadow pipits and field voles are at low densities (2 pipits counted per km of transect, and 0.1 voles caught per 100 trap nights, htn^−1^) , whereas in the right-hand column (c,f), both pipits and voles are abundant (20 pipits.km^−1^, 4 voles.htn^−1^). The middle column (b,e) represents an intermediate case (9 pipits.km^−1^; 1 vole.htn^−1^).

The multi-species model has estimates of *m>1* for all prey species, and the values of *m* also vary between prey species. This is evidence that prey preferences are variable i.e. that switching would be expected in this system. Because of this, and also because our prey abundances are expressed as indices rather than absolute values, we do not attempt to interpret the estimates of *a* directly in terms of prey preference. Instead, we use the Bayesian framework to illustrate the emergent properties of our functional response and its implications for grouse. The grouse component of the MSFR and its implications for harrier-induced grouse mortality at a range of alternative prey densities are shown in Fig. 1, where an index of mortality rate is calculated simply from *F/N* (i.e. the gradient of the functional response curve). Although *m_grouse_≫1* for the MSFR, implying a sigmoidal response to this prey species, the consequences of this are only obvious at low densities of voles and pipits (Fig. 1a), when there is a sharp peak in harrier-induced grouse mortality at low grouse density (Fig. 1d). At higher densities of voles or pipits, the maximum grouse mortality caused by a pair of harriers is around half of its corresponding value when alternative prey are rare.

## Discussion

**Figure 2:**
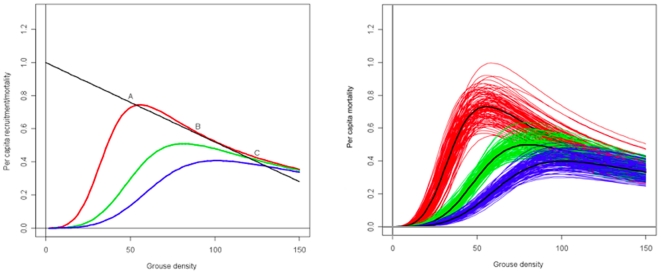
Relationship between the density of grouse chicks, per capita grouse recruitment and per capita chick mortality induced by a population of hen harriers. (a)Harriers were assumed to be present at a density of 0.16 pairs.km^−2^ (which is typical for the study sites [32]). The equilibrium density for the grouse population in the absence of harriers is assumed to be 208 chicks.km^−2^ [35]. Mortality curves are shown for the same three densities of alternative prey as those in Fig. 1 (red = low, green = intermediate, blue = high). Equilibria occur where the mortality curves and the recruitment line intersect. Equilibria A and C are stable, equilibrium B is unstable. (b)Uncertainty in the relationship between harrier-induced chick mortality and chick density. Each curve shows the mortality for one set of parameter values drawn at random from the joint posterior distribution for the MSFR model, thus taking account of any correlation among the parameters.

The impact of a predator on prey population dynamics will depend not only on the MSFR, but also on the predator's numerical response to changes in the density of all prey species [17], [41]. Other mechanisms, such as prey behaviour, may also contribute to the underlying dynamics of the prey population [42]. The possible implications of the MSFR for prey dynamics in a simple system can be investigated graphically [43]. To illustrate this, we plotted per capita grouse recruitment (in the absence of predators), and per capita harrier-induced grouse mortality, against grouse density (Fig. 2a). At grouse densities where these curves intersect, removals due to predation are exactly compensated by the production of new individuals through reproduction – we then expect the prey population to remain constant and these points are referred to as equilibrium grouse densities. The curves indicate these, provided that densities of the predator and other prey (voles and pipits) remain constant. ‘Stable equilibrium’ occurs if predation pressure increases above, and decreases below. This will tend to stabilise the prey population towards the equilibrium point if transient external influences lead to short-term population growth or shrinkage. ‘Unstable equilibrium’ is expected if predation pressure decreases above, and increases below the equilibrium point. In this case, small departures from equilibrium (e.g. due to environmental fluctuations) would be expected to produce dramatic declines or growth in the prey population.

For this illustration, we assumed that grouse density, in the absence of predation, is determined by a simple logistic model. The figure is characterised by one unstable equilibrium (labelled B in Fig. 2a) and two stable equilibria (labelled A and C in Fig. 2a). Equilibrium A is sometimes referred to as a ‘predator pit’. In our system it only occurs at low densities of alternative prey. If the density of alternative prey is increased, only the high grouse density equilibrium (C) appears to be possible. However, the uncertainty associated with the estimates of the MSFR implies that there is a family of possible harrier-induced mortality curves associated with each alternative prey density (Fig 2b). Some members of this family give rise to three equilibria and some to only one. By resampling from the posterior distributions for the parameters of the MSFR, we found that there is 0.56 probability that a predator pit exists when the density of alternative prey is low, but this probability is reduced to 0.06 at intermediate densities of alternative prey and to 0.04 at high densities. This implies, for example, that active habitat management may almost eliminate the existence of the predator pit equilibrium and thereby contribute to more stable high-density grouse populations.

The true nature of the interaction between grouse, hen harriers and alternative prey is likely to be more complex than this. Importantly, while we have assumed that harrier density remains constant, it is known that hen harriers show a multi-species aggregative response (i.e. a numerical response) to all three prey [32], [29]. It is known that the effect of predator numerical and functional responses acting together is important in some systems [44] and this should also be considered in the multi-species context when populations of generalist predators are able to exploit a variety of prey [44]. In the hen-harrier grouse system, the effect of the multi-species aggregative response is to increase the number of harriers when alternative prey is present at intermediate or high densities, and the multi-species functional response then predicts an increase in predation mortality on grouse chicks. The main consequence is that, for intermediate-to-high levels of voles/pipits, the system is likely to have only one stable grouse equilibrium, which – depending on harrier density – may be close to, or below the level observed in the absence of predators. This can be seen as an example of apparent competition, because here the presence of another prey species depresses population levels of grouse due to an indirect effect mediated by a predator. Similar effects have been observed in other mammal populations [45].

A full exploration of the consequences of combined numerical and functional responses for the grouse hen-harrier system is beyond the scope of this paper and requires considerable further analysis that would need to take into account, for example, the rather complex nature of grouse population dynamics [42]. There is good empirical and theoretical evidence that density-dependent population regulation in grouse gives rise to intrinsic population cycles in the absence of predation [46]. The parameterisation of an MSFR that takes account of all important predator prey interactions is a key step towards modelling multi-species population dynamics, improving our understanding of mechanisms operating in this multi-species system and providing parameter estimates that can be used to assist in the process of fitting a full dynamical population model. This may be done by ‘fixing’ MSFR parameters within the dynamical model, thus reducing the number of parameters that must be estimated from time series of population counts [42], [41]. Alternatively, if the entire population model is to be fitted to time-series data using Bayesian methods, a previously fitted MSFR can be used to provide informative priors for its parameters [37].

Our results show that using a single-species FR to model the behaviour of a generalist predator could result in misleading conclusions about the potential effects of that predator on its prey. The fitted form of the single-species FR depends critically on the densities of alternative prey on each occasion that the density and consumption of the focal prey species were measured, and on the assumptions that are made about error distribution. However, the densities of alternative prey weight the generalist predator's estimated single-species FR in unpredictable and potentially highly variable ways. These problems can be overcome by fitting an MSFR, but it is important to have consumption data from a diverse range of prey densities to avoid the need for extrapolation.

Where an MSFR is of interest for an ecological study, field data are more appropriate than those collected in a laboratory situation because of the difficulties involved in realistically replicating prey availability in the laboratory [47]. Multispecies feeding data are usually analysed for evidence of switching between alternative prey [48], but the results of such analyses are hard to interpret quantitatively. Fitting an MSFR requires no more data than an analysis of switching or of frequency-dependent selection, but it provides quantitative information on the predator's behaviour that can be used to predict its consumption over the entire range of observed prey densities. Depending on the nature of available data, it may be possible to use readily available software such as WinBUGS [49] to fit a multi-species functional response [50].

Many different functional response models have been proposed for both single and multi-species systems, and some of these involve biological effects that we have not included in our formulation [28]. For example, competition between predators can be captured by using a measure of the ratio of prey to predators, rather than some absolute measure of prey abundance [51]. Such models have been found appropriate, for example, for mammalian predators in a terrestrial system [52]. However there is evidence to suggest that ratio dependence may not be important in the hen-harrier grouse system [32], and we consider that our formulation for the MSFR is a reasonable choice for our study: it can reproduce all of the standard forms (Type 1, 2 and 3) for a single species FR and does not generate any of the anomalous dynamics shown by some other formulations [28]. It should be noted that the fitting approach presented here can be used with any functional form of MSFR, and Bayesian methodology can potentially be extended further to assess the relative performance of different functional forms, thus accounting for model uncertainty [37].

### Conclusions

Generalist predators have been implicated in a wide range of conservation problems and other conflicts with humans [53]. An understanding of their MSFR, and the uncertainties associated with these responses, is essential for the sound management of pests or endangered species. It is now clear that this can be achieved with a relatively sparse data sets (eg [50]). The MSFR also provides a much-needed link between models of individual predator-prey interactions and ecosystem models.
